# Label-free tumor cells classification using deep learning and high-content imaging

**DOI:** 10.1038/s41597-023-02482-8

**Published:** 2023-08-26

**Authors:** Chawan Piansaddhayanon, Chonnuttida Koracharkornradt, Napat Laosaengpha, Qingyi Tao, Praewphan Ingrungruanglert, Nipan Israsena, Ekapol Chuangsuwanich, Sira Sriswasdi

**Affiliations:** 1 Department of Computer Engineering, Faculty of Engineering, Chulalongkorn University, Bangkok, 10330 Thailand; 2 Center of Excellence in Computational Molecular Biology, Faculty of Medicine, Chulalongkorn University, Bangkok, 10330 Thailand; 3 Chula Intelligent and Complex Systems, Faculty of Science, Chulalongkorn University, Bangkok, 10330 Thailand; 4 NVIDIA AI Technology Center, Singapore, Singapore; 5 Center of Excellence for Stem Cell and Cell Therapy, Faculty of Medicine, Chulalongkorn University, Bangkok, 10330 Thailand; 6 Department of Pharmacology, Faculty of Medicine, Chulalongkorn University, Bangkok, 10330 Thailand; 7 Center for Artificial Intelligence in Medicine, Research Affairs, Faculty of Medicine, Chulalongkorn University, Bangkok, 10330 Thailand

**Keywords:** Machine learning, Cellular imaging

## Abstract

Many studies have shown that cellular morphology can be used to distinguish spiked-in tumor cells in blood sample background. However, most validation experiments included only homogeneous cell lines and inadequately captured the broad morphological heterogeneity of cancer cells. Furthermore, normal, non-blood cells could be erroneously classified as cancer because their morphology differ from blood cells. Here, we constructed a dataset of microscopic images of organoid-derived cancer and normal cell with diverse morphology and developed a proof-of-concept deep learning model that can distinguish cancer cells from normal cells within an unlabeled microscopy image. In total, more than 75,000 organoid-drived cells from 3 cholangiocarcinoma patients were collected. The model achieved an area under the receiver operating characteristics curve (AUROC) of 0.78 and can generalize to cell images from an unseen patient. These resources serve as a foundation for an automated, robust platform for circulating tumor cell detection.

## Background & Summary

Circulating tumor cell (CTC), or cell from primary tumor that were shed into the patient’s bloodstream, holds important clinical values as a source of early, non-invasive biomarker of metastasis and cancer prognosis and many cancer types^1,2^. Existing technologies for isolating and detecting CTC mainly rely on the fact that most normal blood cells can be captured by antibody targeting certain cell surface markers, such as CD45, while tumor cells can be captured by antibody targeting different markers^3^. Although multiple antibodies have been developed for characterizing various CTC types, such as epithelial and mesenchymal CTC^4^, enrichment-based approaches still cannot account for the full heterogenicity of CTC. In fact, a study of lung cancer patients has shown that only 40–60% of CTC in blood samples were detected by enrichment-based approaches^5^. Nowadays, high-throughput sequencing technologies have also been applied to characterize the genome and transcriptome of individual CTC^6^ as a non-invasive mean to probe the molecular signature of primary tumors and to develop prognostic cancer biomarkers.

Another possibility for unbiased characterization of individual CTC is through high-content microscopy imaging of patient blood samples, whereby cancer cells can be differentiated from normal cells as well as classified into types based on their distinctive morphological properties^7,8^. These techniques are enabled by recent advances in deep learning which let us train artificial neural network models to accurately identify cell types^9,10^ and pinpointing the locations of subcellular compartments^11,12^ from bright-field microscopy images without any labeling of the cells. A recent work has also shown that CTCs derived from different tumor sites exhibit clearly distinct morphological characteristics^13^. This suggests the possibility of simultaneously detecting and predicting the tissue-of-origin for each CTC.

However, imaging-based CTC detections were mostly developed and/or validated only on spiked-in cells from a few cell lines that do not capture the broad heterogeneity and morphological properties of actual CTC^14^. For example, Wang *et al*.^15^ trained a deep learning model using 436 cultured cells and 1,309 white blood cells and validated their model on 32 CTCs from two patients. Although Guo *et al*.^16^ trained a deep learning model on 555 CTCs and 10,777 non-CTCs from 776 patients, the technique relied on counting the copy number of chromosome 8 via CEP8 immunofluorescence labeling instead of cell morphology. Other large-scale cell image datasets suitable for developing deep learning models are also similarity restricted to the morphology of cells from established cell lines^17,18^. Hence, the first step toward developing a generalized imaging-based CTC detection platform is to establish a large-scale microscopy imaging dataset of cancer and normal cells that capture the heterogeneity of both cancer types and tissue types.

Patient-derived organoids, or 3D cultures, have been shown as realistic sources of diverse cell types and morphology that faithfully represent the genotype and phenotype of cancer subtypes^19,20^. The combination of paired cancer and normal cells derived from the same tissue of the same patient would serve as a good benchmark for an imaging-based CTC detection technique by testing whether the technique can distinguish between cancer and normal cells (as supposed to distinguishing between blood and non-blood cells). By expanding the dataset of cell images to cover multiple tissues, cancer types, and patients, and by linking cell images to prognosis and treatment response information, future imaging-based CTC platforms have the potential to not only detect CTC, but also predict the tissue-of-origin and aid clinical decision making.

In addition to acquiring more realistic data, improving the cancer cell detection model’s ability to handle imaging artefacts and cluttering of cells is also an important consideration for real-world applications. Past studies^13,15,16,21^ mostly focused on the model’s ability to classify whether a small proposed image regions contain a cancer cell (a classification task), but not necessarily the model’s ability to identify cancer cell locations in a large image with densely populated objects (a detection task). In pathological imaging domains^22,23^, multi-stage deep object detection pipelines have been widely and successfully utilized to address the issue of interfering artefacts and overlapping cells in cell detection tasks. Hence, a similar approach may be beneficial for cancer cell detection.

In this research, a large dataset of microscopic images of more than 75,000 individual organoid-derived cancer and normal cells from 3 cholangiocarcinoma patients were constructed, and a proof-of-concept deep neural network model was developed to (i) evaluate the possibility of distinguishing cancer and normal cells based on only unlabeled bright-field microscopic images and (ii) explore the morphological diversity of cancer and normal cells across cancer types and individual patients. Similar to recent efforts to catalog the molecular heterogeneity of organoids at single-cell resolution^24^, our work contributes to the frontier of cellular morphology resources in the same fashion. Furthermore, our dataset significantly expands existing collections of brightfield organoid cell images by several folds^25,26^. The full dataset and code used for development are available at Figshare^27^ and https://github.com/cmb-chula/CancerCellVision-CCA, respectively.

## Methods

### Cholangiocyte organoid culture

Human liver tissues were obtained from patients undergoing surgery. The use of human cells for research in this study was approved by the Internal Review Board of the Faculty of Medicine, Chulalongkorn University (IRB No. 331/63). Informed contents were obtained from all patients.

For organoid establishment, liver tissues were cut into small pieces and washed 3 times with Advanced DMEM/F12 supplemented with 1x Glutamax, 10 mM HEPES, and 1x antibiotics (AdDF+++, Gibco, Thermo Scientific). Liver tissues were digested using 100 *μ*g/ml dispase I and 300 U/ml collagenase XI in Cholangiocyte culture media with Advanced DMEM/F12 containing 10% R-Spondin condition media, 10% Wnt3a condition media, 1 mM N-Acetylcysteine, 10 mM Nicotinamide, 1x B27 supplement, 1x N2 supplement, 100 ng/ml Noggin, 10 nM Gastrin-I, 50 ng/ml EGF, 5 uM A83-01, 100 ng/ml FGF10 (Peprotech), 25 ng/ml HGF (R&D Systems), and 10 *μ*M FSK (Tocris). The cultures were incubated at 37 °C for 1 hour. The digestion reaction was stopped with 10 ml AdDF+++ and the resulting suspension was filtered through a 70 *μ*M cell strainer. Cells in suspension were collected via centrifugation and washed 5 times with AdDF+++. Cell pellets were resuspended in 70% Matrigel (Corning) and dropped on pre-warmed 24-well culture plates. After the Matrigel solidified, 500 *μ*l of organoid culture media was added. Cells were cultured at 37 °C with 5% CO2. The media were changed every 3 days and the cell passage was performed every 1-2 weeks by mechanically dissociating the cells with P1000 pipette tip.

### Fluorescence labeling and high-content imaging

Each organoid was dissociated into single cells using TrypLETM Express Enzyme (Gibco, Thermo Scientific). Around 106 cancer and normal cells were obtained from each sample. Cells from cancer organoids were stained with a deep red fluorescence (Cytopainter ab176736) while cells from normal organoids were stained with green fluorescence (Cytopainter ab176735). Nuclei were stained with Hoechst. Cancer and normal cells were mixed at 1:1 ratio, dropped on 96-well plates, and subjected to bright-field and fluorescence imaging on an Opera Phenix instrument (Perkin Elmer). In total, 1207 paired bright-field and fluorescence images were acquired for cancer and normal cholangiocytes. Each image consists of 1080 × 1080 pixels and contains 20–30 individual cells on average.

### Image processing and preparation

**Fig. 1:**
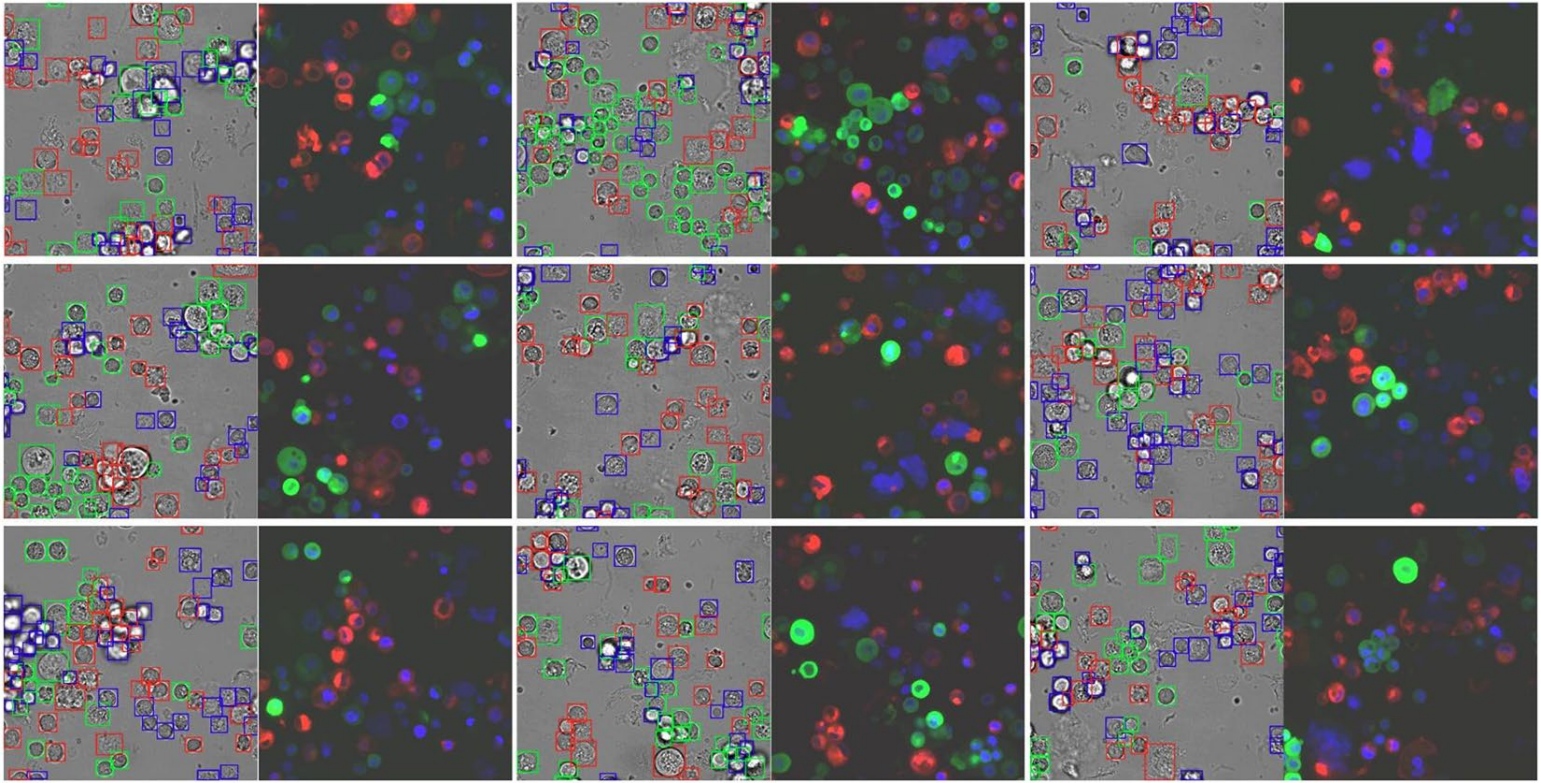
Examples of preprocessed and annotated brightfield and fluorescence image for human annotator. Box colors indicate the object classes (red for cancer cells, green for normal cells, and blue for unknown cells that exhibited neither signals).

Prior to the annotation step, brightfield and fluorescence images were prepossessed to make the individual cells more visually distinguishable to the human eyes. Data preprocessing steps described in Christiansen *et al*.^11^ were performed with some modification. First, a median filter of size 5 × 5 was repeatedly applied to the fluorescence images until convergence to reduce the salt-and-pepper noise. After that, images were bilinearly downsampled by a factor of two to reduce shot noise. Finally, pixel intensities were normalized per image to the same mean and standard deviation. Frame stitching did not need to be performed due to the difference in data acquisition technique. Flat field correction and dust artifact removal were also not applied because these operations did not significantly affect the quality of images here. After preprocessing, the three fluorescence activations (red for cancer cells, green for normal cells, and Hoechst blue for nuclei) of each image were merged into a single three-channel image. Examples of prepossessed and annotated images are shown in Fig. 1.

### Cell annotation

**Fig. 2:**
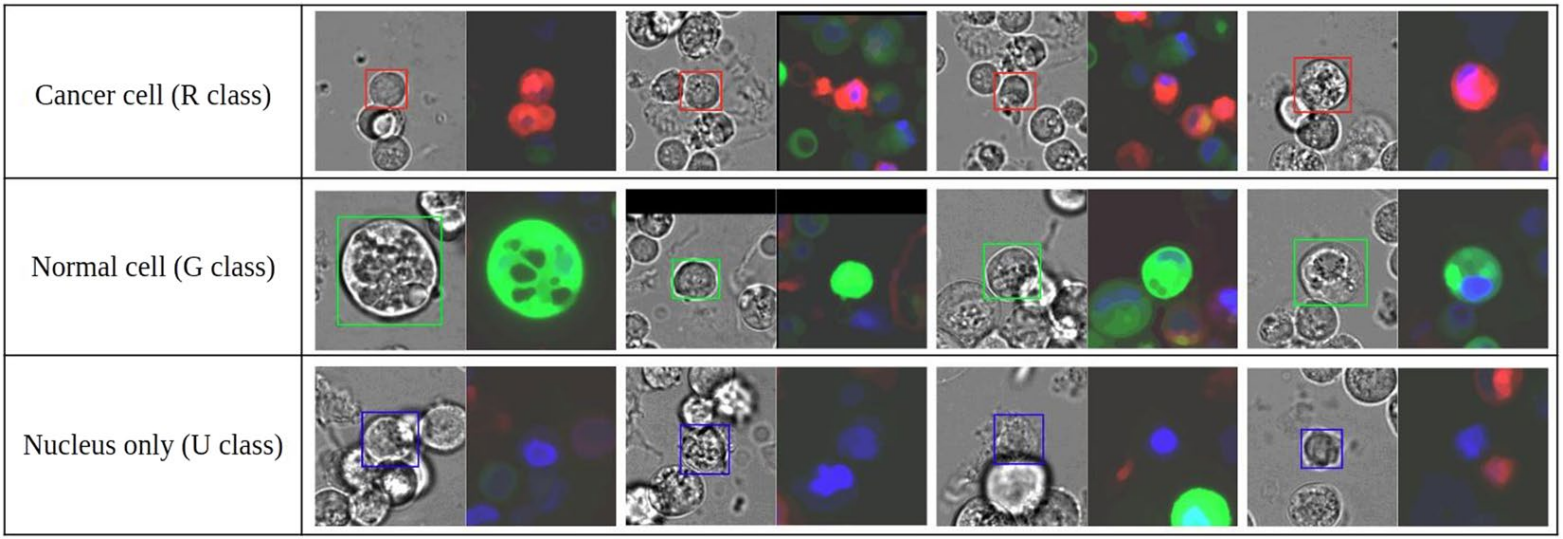
Examples of annotated cells from each class.

There were three human annotators. One annotator is an expert in microscopy with more than three years of experience. The other annotators are graduate biology students. An inter-annotator agreement was evaluated at the beginning by asking all three annotators to analyze the same set of 6 images (about 150 individual cells). Labelme^28^ was used to annotate the location and classification of each cell. Brightfield image and the corresponding fluorescence image were simultaneously shown to the annotators. Cells were classified as either cancer, if there was a clear red fluorescence signal, normal, if there was a clear green fluorescence signal, or unknown, if only the Hoechst signal was visible (Fig. 2).

**Fig. 3:**
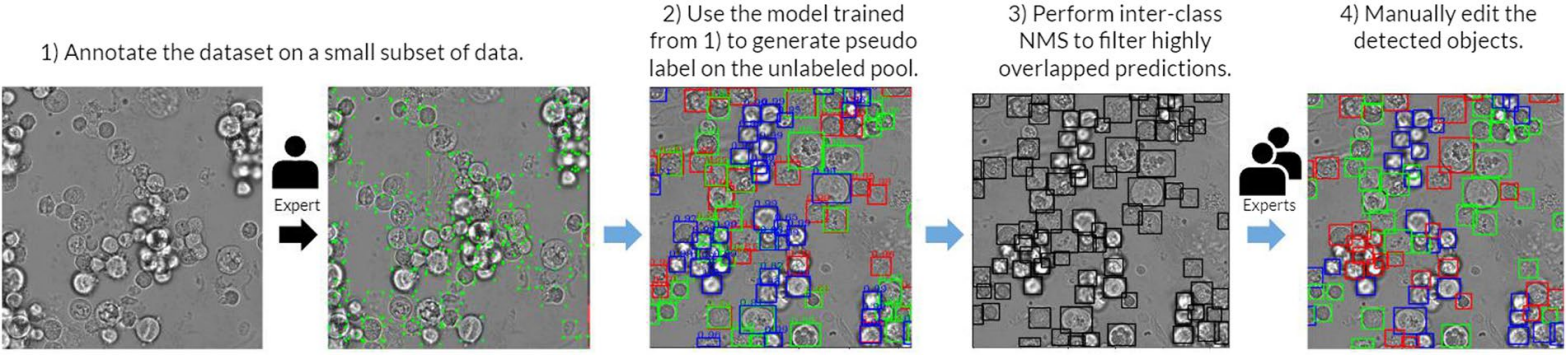
The annotation process for the training and validation sets. First, a small subset (30 images) was fully manually annotated. Then, the initial cell detection and classification model was trained to generate pseudolabels for all unannotated images. The pseudo-generated bounding boxes were then filtered using Non-Maximum Suppression (NMS) to remove highly overlapping boxes. These pseudolabel annotation were then refined by the experts to obtain the final annotation used for training and validation. Note that every step in this annotation process used fluorescence images as guidance.

**Fig. 4:**
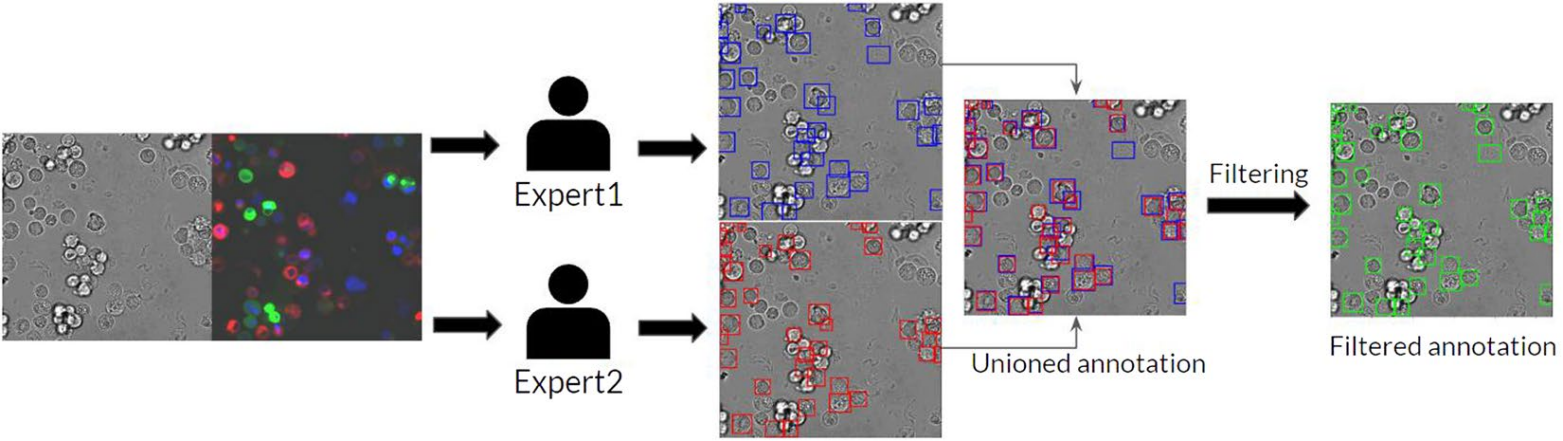
The annotation process for the test set. Two annotators were separately tasked to annotate all cancer cells inside each brightfield image with paired fluorescence image as guidance. Results from the two annotators were combined and used as the final annotation.

The annotation process were divided into three phases (Figs. 3, 4). In the first phase, a subset of 30 images were fully annotated by the most experienced annotator and then used to train an initial object detection model, with both brightfield and fluorescence images as inputs. In the second phase, the initial model was used to generate bounding boxes and classification for the remaining images and the results were provided to the annotators for further refinements. Annotators can add new bounding boxes, remove erroneous bounding boxes, or change the classification of each cell. At the end of the second phase, 1087 out of 1207 images were analyzed by at least one annotator. These data were used to train the proof-of-concept model.

In the third phase, to construct the test set, 120 images were sampled from three patients (40 images each) and manually annotated by human annotators. Bounding boxes and classification labels from the initial object detection model were intentionally withheld to minimize biases. Furthermore, to maintain high annotation quality, each image was analyzed by at least two annotators and only cancer cells were annotated. The bounding boxes defined by the two annotators were merged using non-maximum suppression (NMS). When there is disagreement, bounding boxes produced by the annotator with more experience were used.

## Data Records

The dataset consists of 1207 paired brightfield and fluorescence microscopy images with a resolution of 1080 × 1080 in the TIFF format with cell-level bounding box and classification annotations in the VOC format. The dataset is available on FigShare^27^. There are 84,503 cell-level bounding box annotations consisting of a bounding box (xmin, ymin, w, h), and object class. The three object classes are R, G, and U, which refer to tumor cell (red fluorescence), normal cell (green fluorescence), and unknown cell, respectively. The dataset is separated into training, validation, and test splits, where the test split contains only cancer cell annotation, while the rest have all three classes. The number of objects from each class in each data split is shown in Table 1.

**Table 1:** The number of images and cells in each dataset split.

Data split	No. of image	R	G	U
Train	967	22800	24862	24575
Validation	120	2823	2972	3135
Test	120	3336	(3374)	(3459)

Numbers in brackets were estimated from model prediction results under the guidance of fluorescence signal. For reference, the average precision for R, G, and U class on the validation split is 89.2, 88.0, and 80.8, respectively.

### Detailed description

**Fig. 5:**
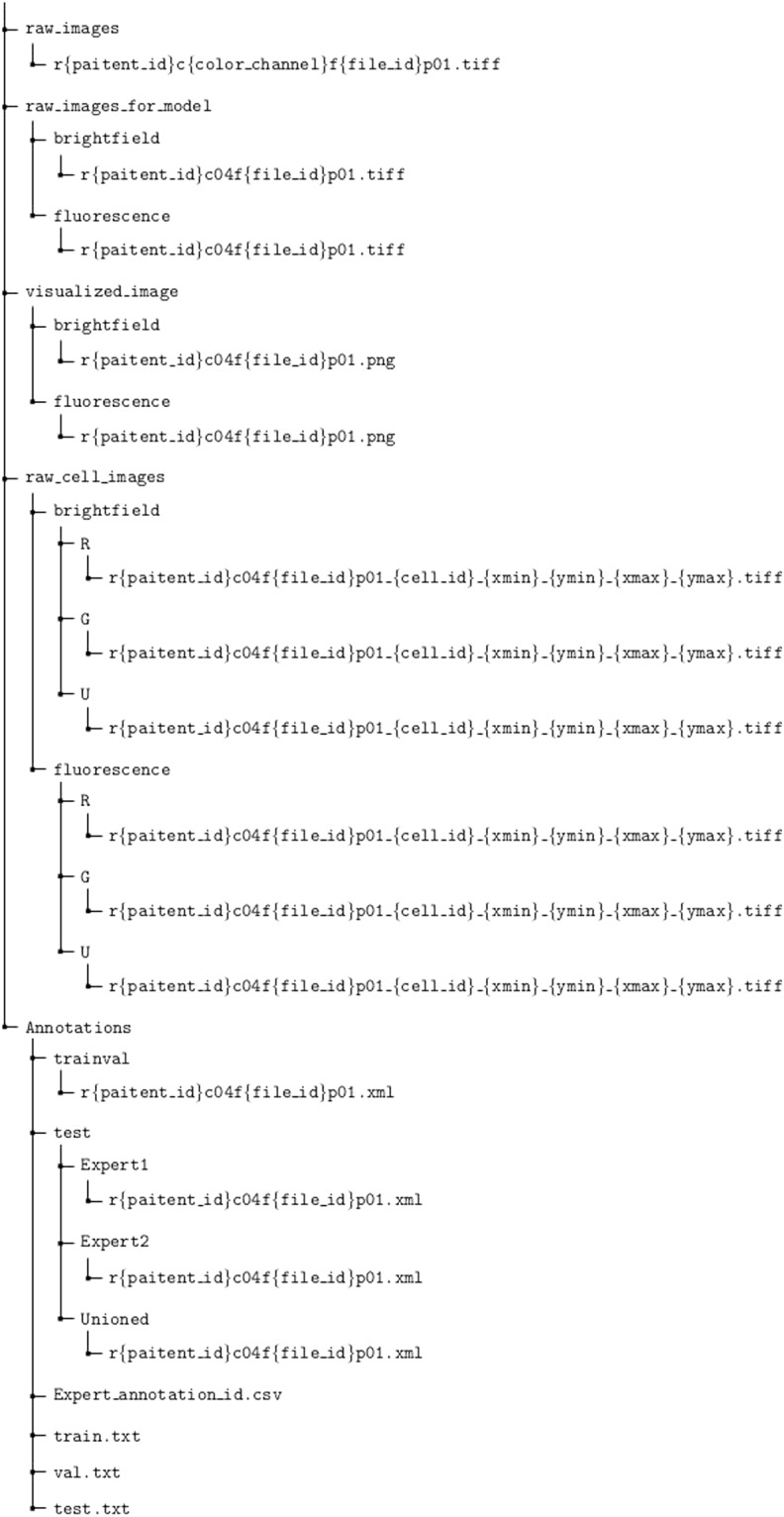
The index of our proposed dataset.

Figure 5 summarizes the indexing structure of our dataset. Original raw image files are stored in the directory raw_images_for_model. This directory consists of two sub-directories: raw_images_for_model/brightfield contains brightfield images and raw_images_for_model/fluorescence contains fluorescence images. Files are named with the r{patient_id}c04f{file_id}p01.tiff format, where patient_id and file_id refers to the IDs of the patients (06, 07 or 08) and image, respectively. Each brightfield image and the corresponding fluorescence image share the same file name. Each fluorescence image is a three-channel image file where channels correspond to red fluorescence signal (cancer cells), green fluorescence signal (normal cells), and Hoechst signal (nuclei), respectively. These raw images can be readily used as input for the detection stage without further post-processing.

Annotations are provided in the directory Annotations. Annotations for the training-validation split and the test split are provided separately in subdirectories trainval and test, respectively. Each annotation file is named with the same r{paitent_id}c04f{file_id}p01.xml format as the raw image files provided in raw_images_for_model. The test subdirectory contains three subdirectories: Expert1, Expert2, and Unioned, which contain the annotations from the first expert, second expert, and the combined version, respectively.

Images of individual extracted cells, which are ready to use for cell classification, are provided in the directory raw_cell_images. There are three subdirectories R, G and U, each containing images of cells from each class. The file name of each cell follows the r{paitent_id}c04f{file_id}p01_{cell_id}_{xmin}_{ymin}_{xmax}_{ymax}.tiff format, where cell_id refers to the ID of each cell, and (xmin, ymin, xmax, ymax) indicates the position of the cell in the raw image r{paitent_id}c04f{file_id}p01.tiff.

Each line in train.txt, val.txt, and test.txt indicates the split of each data point. The file expert_annotation_id.csv contains the ID of the annotator who analyzed each image.

## Technical Validation

Technical validations of our dataset were conducted by training a deep learning model to recognize cancer cells in given brightfield (unlabeled) microscopy image. Evaluations were performed at two levels: cell level and image level. The cell-level evaluation measures the model’s ability to distinguish between cancer (class R) and other cell types (classes G and U) from given cropped cells from the brightfield image as an input. On the other hand, the image-level evaluation measures the model’s ability to do so on the whole brightfield image. This setup introduces additional challenges since the model also has to differentiate cancer cells from background objects and imaging artifacts.

The experiments were conducted under three input settings: Brightfield, Brightfield + Hoechst, and Brightfield + Fluorescence. The Brightfield setting is a standard setup where the model receives only the brightfield images as an input, while under the Brightfield + Hoechst or Brightfield + Fluorescence settings, Hoechst fluorescence signals or all fluorescence signals were also provided as input, respectively. The Brightfield + Hoechst setting reflects the situation where nuclei staining data are available. The Brightfield + Fluorescence setting was included to evaluate the upper bound of cancer cell recognition performance (as fluorescence signals that contain the ground truth are provided).

**Fig. 6:**
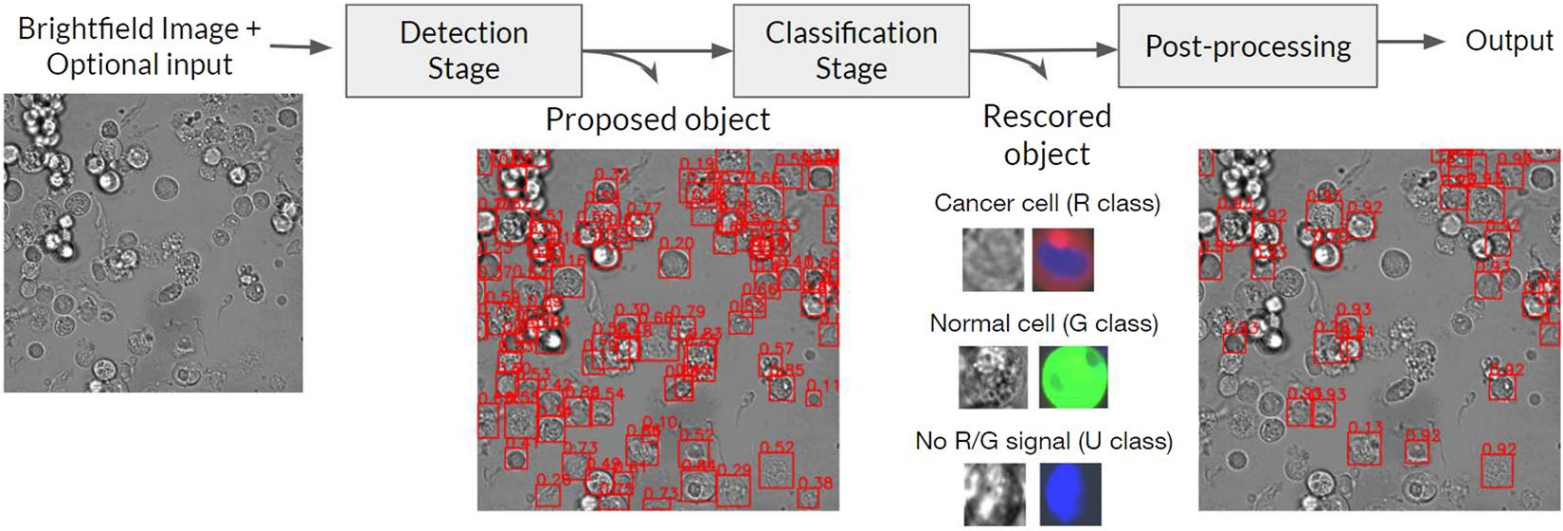
The main pipeline for cancer cell detection consists of two stages, detection and classification, each being a deep artificial neural network. The detector proposes possible cancer cells which are then re-examined by the classifier to refine the confidence scores. Finally, Non-Maximum Suppression (NMS) is performed to remove highly overlapping bounding boxes.

Here, a two-stage detection pipeline consisting of a detector and a classifier was developed. The detector is responsible for proposing bounding boxes of objects of interest, while the classifier refines the confidence score of each proposed bounding box. During the cell-level evaluation, the ground truth bounding box of each object was directly provided to the classifier. An overview of the pipeline is shown in Fig. 6.

### Cell detection from brightfield image

A deep object detection artificial neural network based on Faster R-CNN^29^ with ResNet-50^30^ as a network backbone was developed to propose the locations of all cancer cells in a given brightfield image. The model receives an image as an input and return a set of bounding boxes, {(*x*_1_, *y*_1_, *w*_1_, *h*_1_, *S*_1_),…, (*x*_*n*_, *y*_*n*_, *w*_*n*_, *h*_*n*_, *S*_*n*_)}, where each element of a tuple indicates the center of the predicted cell, the dimension of the predicted cell, and the confidence score for the cancer class, respectively. In our benchmarks, the model was trained to detect only cancer cells, as we found that training the model to simultaneously recognize cells from all three classes dampened the performance.

The original 1080 × 1080 pixels resolution of the brightfield image was used for training. The network backbone was initialized using ImageNet pre-trained weights^31^. Minor modifications were made to adjust the number of output classes and the first convolutional layer. The number of input image channels were adjusted to 4 and 6 accordingly when fluorescence signals are provided as input (the Brightfield + Hoechst and Brightfield + Fluorescence settings). The training framework was based on MMDetection^32^. Specifically, the model was trained using a batch size of 4 and stochastic gradient descent (SGD) as an optimizer. The learning rate was set at 10^−3^ for 32 epochs and then divided by a factor of 10 after 16 and 24 epochs have passed. Only random flip augmentation were performed during training.

### Refinement of cell detection results

Downstream from the object detection network is a classifier, which is a deep convolutional neural network (CNN) that outputs the confidence score for each object predicted by the detector. ConvNext-B^33^ was used as the network backbone with a fixed input resolution of 128 × 128 pixels. The network backbone was initialized using ImageNet^31^ pre-trained weights. The model was trained using a batch size of 64 and Adam as an optimizer. The learning rate was set at 1 × 10^−4^ for 18,000 iterations and then divided by a factor of 10 after 10,000 and 14,000 iterations have passed. Random geometric augmentation, gaussian blur, and random brightness augmentation were performed during training. During the image-level evaluation, the confidence score *S* is the weighted average between the scores produced by the detector, *S*_*det*_, and the classifier, *S*_*cls*_, with the weight *ω*, (*S* = (1 − *ω*)*S*_*det*_ + *ωS*_*cls*_). *ω* was set to 0 during the cell-level evaluation to disregard the contribution from the detector.

### Cell-level performance evaluation

Cell-level evaluation was performed on three different training runs to calculate the mean and standard deviation of each performance metric on the validation split. The cancer class confidence thresholds that yielded the highest F1 scores were selected for calculating the precision and recall values. The areas under the receiver operating characteristics curve (AUROCs) were also reported.

**Fig. 7:**
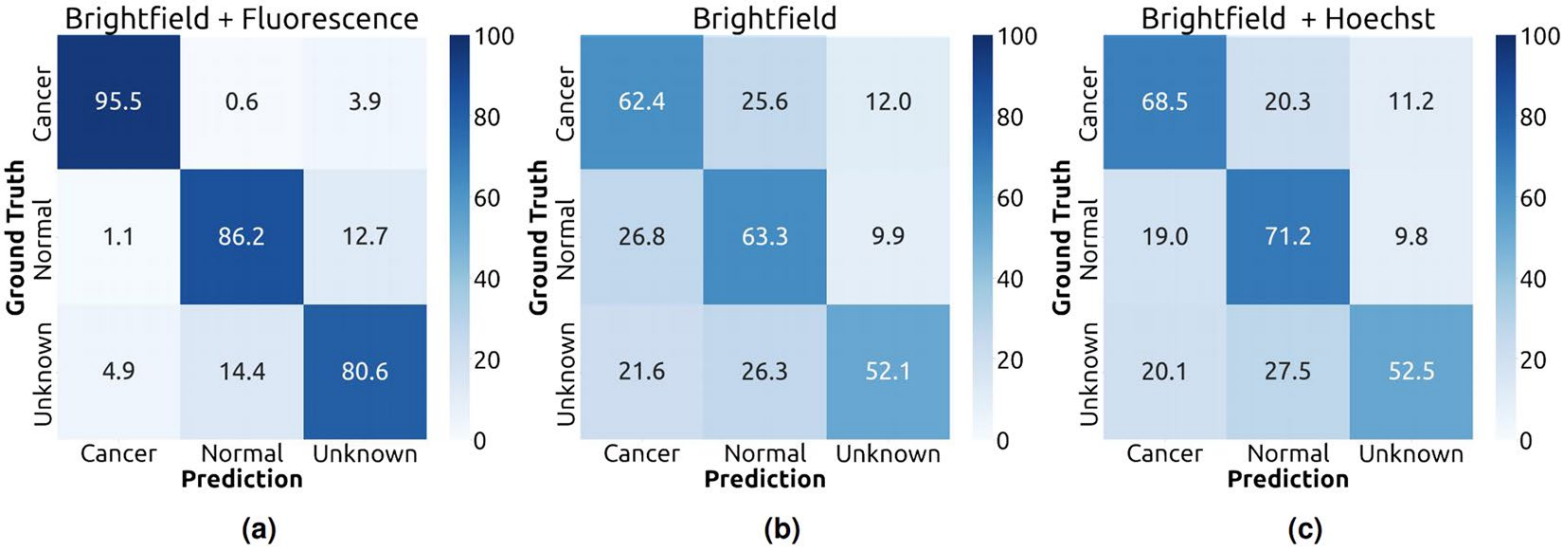
Normalized confusion matrix of the cell-level evaluation on the validation split.

Table 2 summarized the cell-level performance of our model. Unsurprisingly, when both brightfield and fluorescence images were used as input (the Brightfield + Fluorescence setting), the model could accurately recognize cancer cells with an F1 score of 94.5. While this setting is unrealistic, it confirmed the quality and consistency in the annotations. Figure 7a shows that most of the confusions involved unknown cells, which are either cancer or normal cells that exhibit nuclear staining fluorescence but no cytoplasmic staining fluorescence. There was only around 1% confusion between normal and cancer cells. With only brightfield images as input (the Brightfield setting), the cancer cell classification performance dropped to 60.5% F1 with more than 20% confusion between normal and cancer cells (Fig. 7b). When the Hoechst fluorescence channel which indicate the nuclei was included as an input, the classification performance improved noticeably to 66.0% F1 (Fig. 7c). This indicates that the model can take advantage of the differences in nuclear morphology between normal and cancer cell^34^.

**Table 2:** Cell-level cancer classification performance of our method on the validation split of our dataset.

Setting	F1	precision	recall	AUROC
Brightfield	60.5 ± 0.4	50.5 ± 0.6	75.5 ± 1.7	77.5 ± 0.2
Brightfield + Hoechst	66.0 ± 0.2	58.7 ± 0.9	75.5 ± 1.3	83.0 ± 0.2
Brightfield + Fluorescence	94.5 ± 0.1	93.5 ± 0.3	95.5 ± 0.1	99.3 ± 0.1

**Fig. 8:**
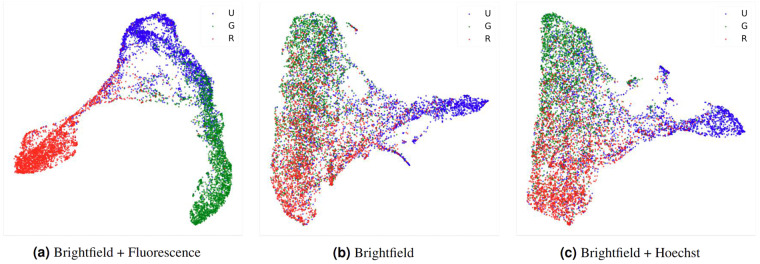
2D embeddings of cells from different classes in the dataset. The embeddings were calculated using UMAP from the feature map at the last layer before the last global average pooling in the network. (**a**) Embeddings from the model trained with brightfield images and all fluorescence signals. (**b**) Embeddings from the model trained using only brightfield images. (**c**) Embeddings from the model trained with brightfield images and Hoeschst signal.

UMAP visualization^35^ of the latent embedding vectors, extracted from the feature map of the last layer before the last global pooling in the neural network, for the individual cells (Fig. 8) shows that unknown cells not only reside between the normal cells and cancer cells but also are visually separable from the other classes. Without full fluorescence information, the learned embeddings were more ambiguous (Fig. 8b,c), especially between normal and cancer cells.

To investigate the impact of neural network architecture choice on cancer cell classification performance, an ablation analysis was conducted by changing the chosen base backbone network (ConvNext^33^) with EfficientNet^36^, DenseNet^37^, ResNet^30^, and Swin Transformer architecture^38^ and their variants. All models used the same training schedule and training configuration as a baseline model, except for Swin Transformer where the training schedule was extended to twice its original duration. Table 3 indicates that a change in network architecture can affect the performances as there were up to 3.0% F1 score and 2.2% AUROC gap between the best and worst performing one, with ConvNext-L achieving the overall highest classification performances.

**Table 3:** The effect of classifier backbone architecture choices on cell-level performances.

Backbone	#Params	Training time	Brightfield		Brightfield + Hoechst		Brightfield + Fluorescence	
			F1	AUROC	F1	AUROC	F1	AUROC
Swin-B^38^	86.7 M	2.80 h	59.9 ± 0.1	75.8 ± 0.1	66.2 ± 0.1	83.0 ± 0.1	94.6 ± 0.1	99.3 ± 0.1
Swin-S^38^	48.8 M	2.18 h	59.8 ± 0.3	77.1 ± 0.1	66.2 ± 0.1	83.0 ± 0.1	94.6 ± 0.1	99.3 ± 0.1
ConvNext-L^33^	196.2 M	1.30 h	61.0 ± 0.1	77.9 ± 0.2	66.4 ± 0.4	83.2 ± 0.2	94.5 ± 0.1	99.3 ± 0.1
ConvNext-B^33^	87.6 M	0.87 h	60.5 ± 0.4	77.5 ± 0.2	66.0 ± 0.2	83.0 ± 0.2	94.5 ± 0.1	99.3 ± 0.1
ConvNext-S^33^	49.5 M	0.75 h	60.2 ± 0.1	77.2 ± 0.1	65.8 ± 0.3	82.7 ± 0.2	94.6 ± 0.1	99.3 ± 0.1
EfficientNet-B7^36^	63.8 M	1.30 h	60.3 ± 0.4	77.1 ± 0.2	65.1 ± 0.2	81.9 ± 0.2	94.5 ± 0.2	99.1 ± 0.2
EfficientNet-B4^36^	17.6 M	0.81 h	60.0 ± 0.1	77.3 ± 0.2	65.2 ± 0.3	82.2 ± 0.1	94.4 ± 0.2	99.2 ± 0.1
EfficientNet-B1^36^	6.5 M	0.71 h	59.1 ± 0.2	76.2 ± 0.2	63.4 ± 0.3	80.6 ± 0.1	94.4 ± 0.2	99.2 ± 0.1
DenseNet-201^37^	18.1 M	1.23 h	60.0 ± 0.4	76.6 ± 0.2	64.6 ± 0.3	81.7 ± 0.3	94.5 ± 0.2	99.3 ± 0.1
DenseNet-169^37^	12.5 M	0.94 h	59.5 ± 0.2	76.5 ± 0.2	64.8 ± 0.2	81.8 ± 0.1	94.2 ± 0.1	99.2 ± 0.1
DenseNet-121^37^	7.0 M	0.75 h	59.3 ± 0.4	76.0 ± 0.3	64.2 ± 0.3	81.1 ± 0.2	94.4 ± 0.4	99.1 ± 0.1
ResNet-152^30^	58.2 M	1.03 h	58.8 ± 0.2	75.7 ± 0.3	63.9 ± 0.3	81.1 ± 0.2	94.5 ± 0.1	99.2 ± 0.1
ResNet-101^30^	42.5 M	0.78 h	59.1 ± 0.4	75.7 ± 0.1	63.6 ± 0.3	80.8 ± 0.1	94.4 ± 0.2	99.2 ± 0.1
ResNet-50^30^	23.5 M	0.55 h	59.1 ± 0.2	75.8 ± 0.1	63.8 ± 0.2	80.9 ± 0.1	94.6 ± 0.1	99.3 ± 0.1

Every experiment was conducted using NVIDIA RTX 3090 and Intel(R) Core(TM) i9-9900K CPU @ 3.60 GHz.

### Image-level performance evaluation

**Fig. 9:**
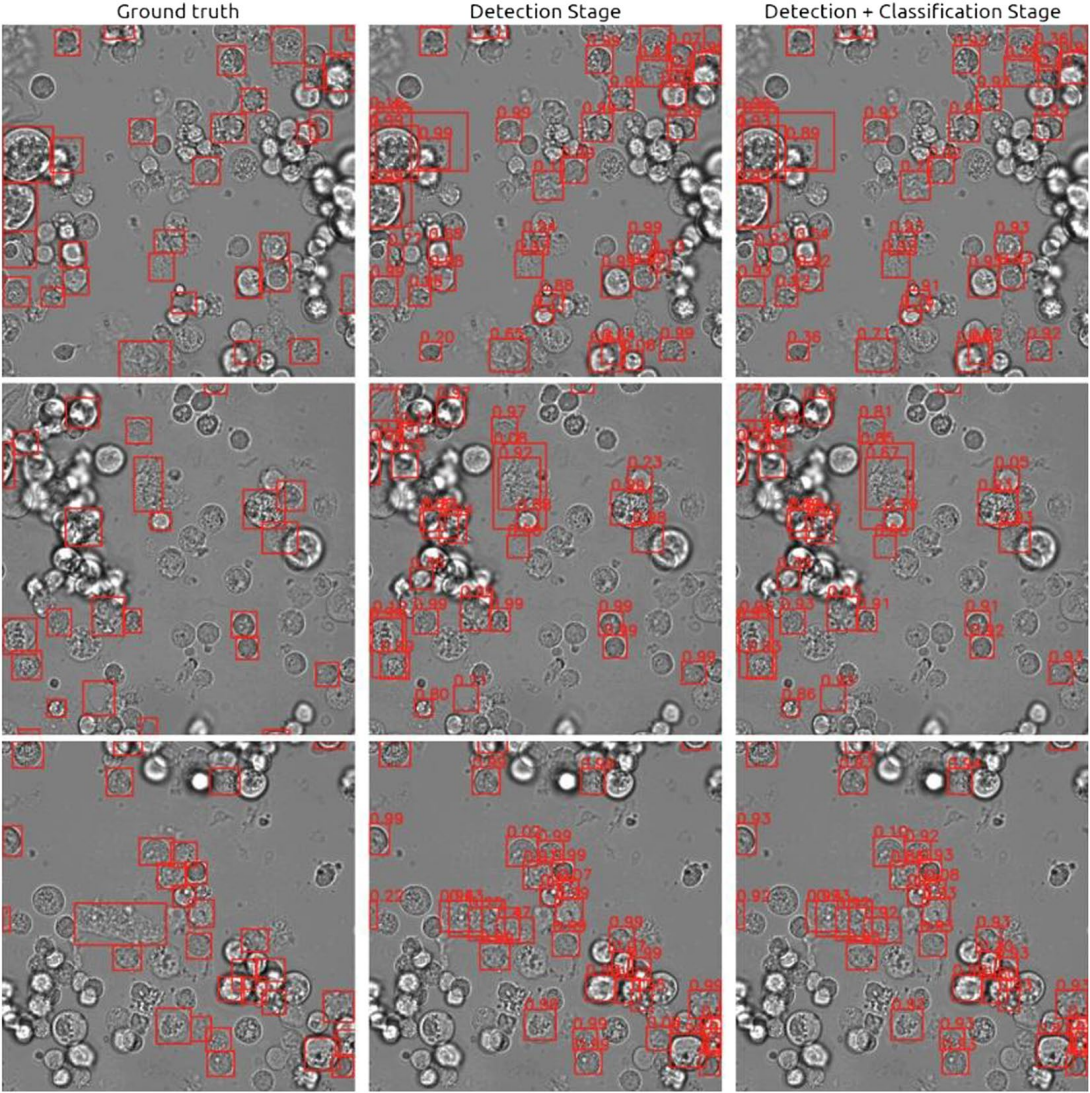
Example of image-level predictions (red boxes) and their confidence on the test set under the Brightfield + Fluorescence setting. Despite having the fluorescence signal as guidance, the model still outputted oversized bounding boxes and could not distinguish individual cells in areas with high cell density.

For the image-level evaluation, the ability of the model to locate cancer cells in a large brightfield image is also measured. Each bounding box predicted by the model is considered a match to a cancer cell if it overlaps with the annotated bounding box with an intersection-over-union (IoU) ratio of at least 0.5. Furthermore, because only the cancer cell class is considered here, the average precision at the IoU threshold of 0.5 (AP50) was measured instead of AUROC. F1 scores were also reported for comparison to the cell-level evaluation. Table 4 shows significant performance improvement in both Brightfield and Brightfield + Hoechst settings when the two-stage architecture (full pipeline) was used over the deep object detector (detection stage). This was because the detector can produce high-confidence false positives when many objects overlap with each other, such as in areas with high density of cells. The downstream classification stage can effectively resolve these errors as it observe each proposed object separately. For the Brightfield + Fluorescence setting, the performance did not change much because some of the bounding boxes generated by the detection stage were oversized and did not sufficiently overlap with the ground truth annotation, even though the predicted classes were correct (Fig. 9). It should be noted that a small performance gain can still be achieved by properly weighing the prediction confidences between the detector and the classifier (*ω* = 0.7).

**Table 4:** Image-level cancer cell detection performance of our method on the test split.

Detection Alogrithm	Setting	Detection stage		Full pipeline (*ω* = 1)		Full pipeline (*ω* = 0.7)	
		AP50	F1	AP50	F1	AP50	F1
Faster R-CNN^29^	Brightfield	43.1 ± 0.6	50.3 ± 0.3	50.6 ± 0.2	54.1 ± 0.2	52.2 ± 0.2	55.4 ± 0.3
	Brightfield + Hoechst	45.5 ± 0.7	52.2 ± 0.3	56.5 ± 0.5	57.8 ± 0.4	57.7 ± 0.3	59.3 ± 0.3
	Brightfield + Fluorescence	86.6 ± 0.3	87.3 ± 0.1	86.7 ± 0.3	86.2 ± 0.2	89.2 ± 0.3	87.8 ± 0.1
FCOS^39^	Brightfield	46.7 ± 0.2	51.5 ± 0.2	51.4 ± 0.1	54.9 ± 0.3	52.4 ± 0.1	55.3 ± 0.3
	Brightfield + Hoechst	48.2 ± 0.6	52.8 ± 0.2	57.7 ± 0.4	58.8 ± 0.4	58.7 ± 0.3	59.2 ± 0.8
	Brightfield + Fluorescence	89.8 ± 0.3	86.0 ± 0.1	88.4 ± 0.4	85.2 ± 0.1	90.8± 0.2	86.7 ± 0.1
DINO^40^	Brightfield	43.6 ± 0.3	49.7 ± 0.2	52.1 ± 0.2	54.4 ± 0.3	52.9 ± 0.1	55.1 ± 0.3
	Brightfield + Hoechst	45.1 ± 0.4	50.4 ± 0.3	58.3 ± 0.3	57.8 ± 0.5	58.9 ± 0.2	58.7 ± 0.4
	Brightfield + Fluorescence	91.0 ± 0.3	86.7 ± 0.5	89.0 ± 0.1	85.9 ± 0.2	91.7 ± 0.3	87.5 ± 0.3

To investigate the impact of detection algorithm on cancer cell detection performance, an ablation analysis was conducted by switching the base detection algorithm from Faster R-CNN to FCOS^39^, an anchor-free object detector, and DINO^40^, a set prediction-based object detector. All models used the ResNet-50 backbone and the same training schedule and configuration, except for DINO where AdamW optimizer with an initial learning rate of 10^−5^ was used. It should be noted that DINO significantly benefited from using a COCO pretrained weight (which covers both the encoder and the decoder) over using an ImageNet pretrained encoder weight with a randomly initialized decoder. The results in Table 4 indicated that the choice of object detection algorithm significantly impacts detection F1 performance. Nevertheless, the difference in performance diminished when the full pipeline with a classification stage and weighted confidence technique was used.

One interesting result is how information from unknown cells (those with unclear cytoplasmic fluorescence signals) could be used to improve cancer cell detection performance. As shown in Table 5, dropping all unknown cells from the training data resulted in a suboptimal F1 of 56.3%. Thus, we performed semi-supervised learning by predicting pseudolabels for unknown cells and adding them to the training set. However, the performance dropped regardless of whether all pseudolabels were included or even when only high-confidence pseudolabels were considered. Curiously, the best improvement with 3.0% additional F1 was achieved by labeling all unknown cells as non-cancer. This is unexpected because there are many unknown cells whose latent embeddings, which reflect the cells’ morphological characteristics, were similar to cancer cells’ (Fig. 8). These unknown cells are expected to be poorly stained cancer cells. A possible explanation is that because the majority of unknown cells are morphologically distinct from both cancer and normal cells (Fig. 8), they might include non-cell objects such as dead cells and other debris. Hence, by treating all unknown cells as non-cancer, the model might better delineate the morphological boundary of cancer cells.

**Table 5:** Impact of various strategies for adding unknown cells to the training set on cancer cell detection performance.

Method	Test F1
Not including unknown cells	56.3 ± 0.2
Assign pseudo labels to all unknown cells	55.2 ± 0.3
Assign pseudo labels to only unknown cells with >0.8 confidence of being cancer	55.2 ± 0.1
Assign all unknown cells as normal	59.3 ± 0.3

The experiments were conducted under the Brightfield + Hoechst setting with confidence weighting (*ω* = 0.7) during inference. The confidence of each pseudolabel was obtained from an average of three inference runs from the models of different seeds.

### Evaluation of patient-to-patient variation

**Fig. 10:**
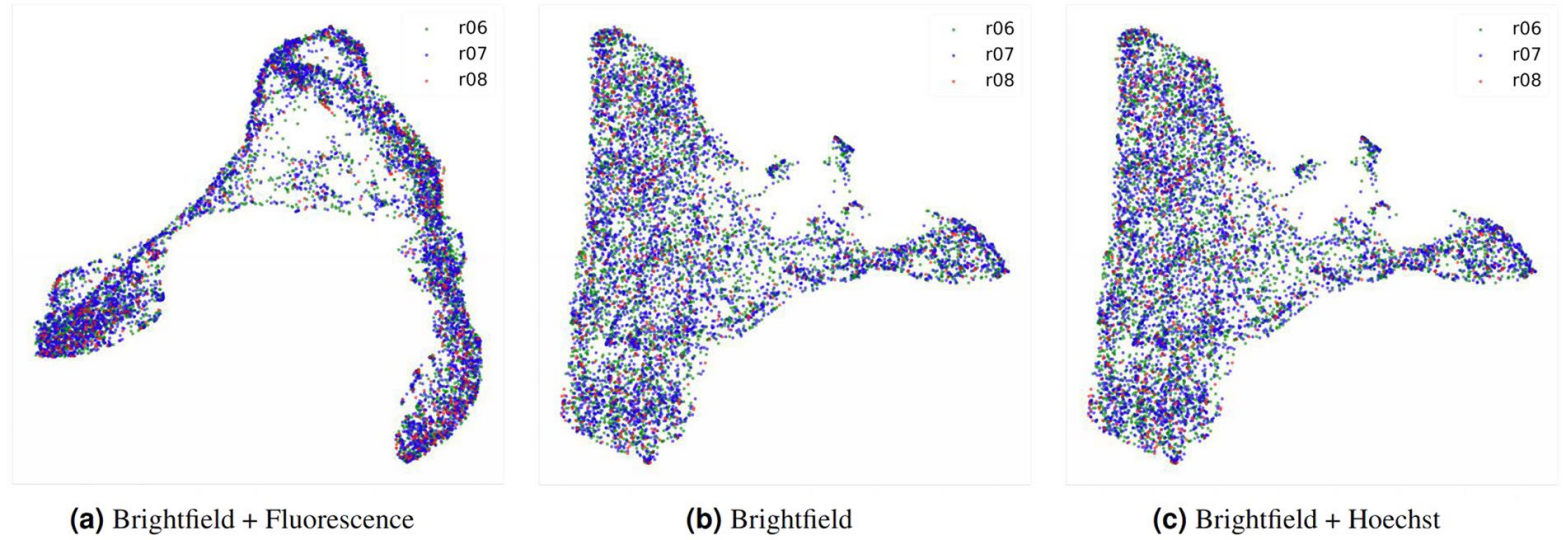
2D embeddings of cells from different patients in the dataset. The embeddings were calculated using UMAP from the feature map at the last layer before the last global average pooling in the network. (**a**) Embeddings from the model trained with brightfield images and all fluorescence signals. (**b**) Embeddings from the model trained using only brightfield images. (**c**) Embeddings from the model trained with brightfield images and Hoeschst signal.

The extent of patient-to-patient variation in cell morphology was evaluated by training the model using data from one or two patient(s) and measuring the performance on data from the unseen patient(s). Overall, the model can generalize to cell images from unseen patients with less than 2% drop in F1 (Table 6). The 2D embeddings of cells from different patient are also similarly distributed (Fig. 10). Although the performances were lowest when the models were trained or tested on data from the third patient, this may be due to small number of annotated images from this patient. In contrast, around 500 images were annotated each for the other two patients. It should be noted that even though the model was able to generalize across the three patients, the same level of performance would not be expected when applying the model to cells from patients of a different population or cells from patients with different underlying molecular causes of cholangiocarcinoma. However, our model weight and dataset should still be useful in a transfer learning framework, where future users can fine-tune the model on their local datasets instead of having to train a new model from scratch. Furthermore, the fact that the model can at least generalize across local patients is a good sign that cell morphology does not vary significantly across individuals.

**Table 6:** Model performances (F1) when trained and tested on cell images from different cholangiocarcinoma patients.

Training data	Number of annotated images	1^*st*^ patient	2^*nd*^ patient	3^*rd*^ patient
All 3 patients	967	60.1 ± 0.2	59.2 ± 0.2	59.4 ± 0.5
1^*st*^ patient	493	58.3 ± 0.2	58.3 ± 0.5	57.9 ± 0.5
2^*nd*^ patient	503	57.7 ± 0.2	56.9 ± 04	58.1 ± 0.4
3^*rd*^ patient	91	39.2 ± 0.4	39.7 ± 0.4	39.2 ± 0.7

The experiments were conducted under the Brightfield + Hoechst setting with confidence weighing (*ω* = 0.7) during inference.

### Impact of dataset size on cancer cell classification

**Fig. 11:**
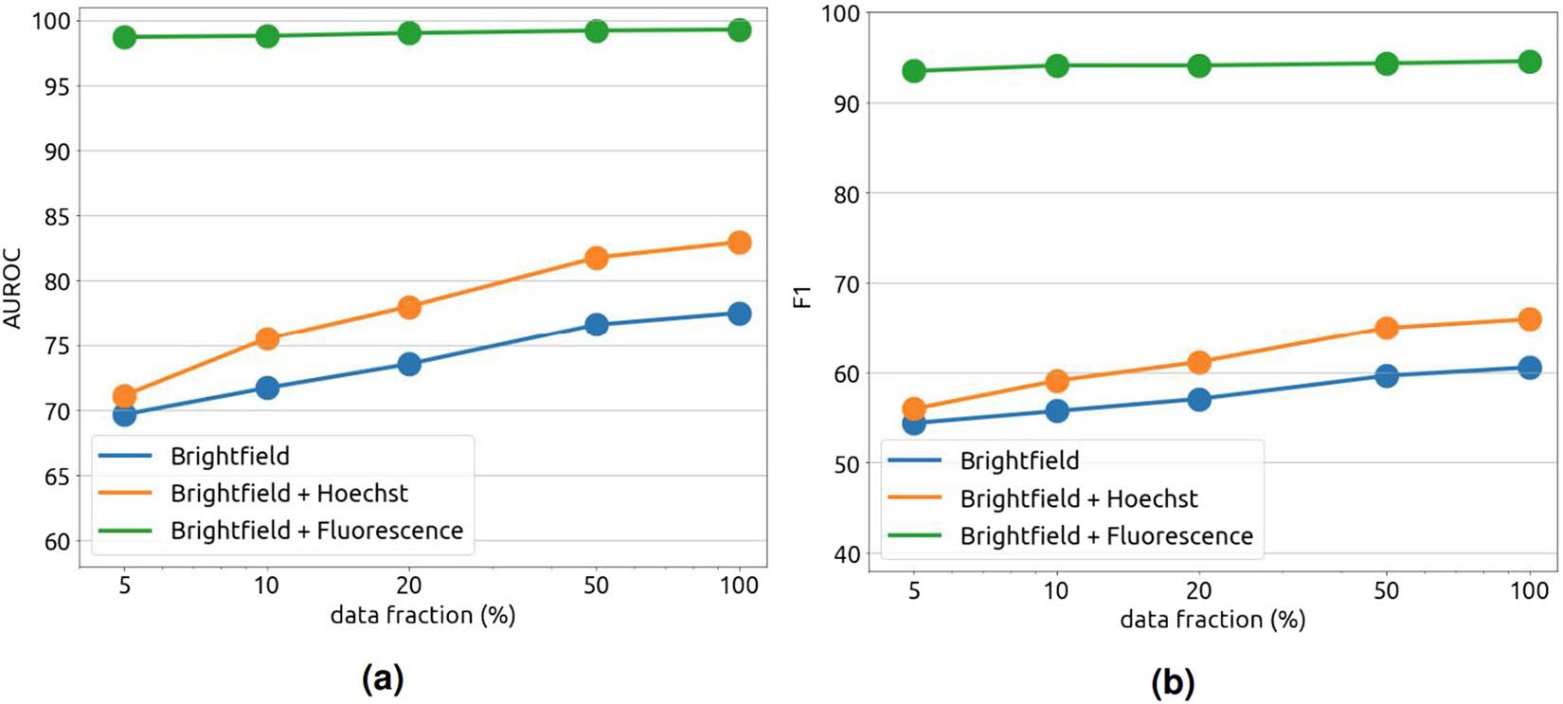
Impact of the training set size on the AUROC and F1 performances of cancer cell classification. Performances of the classifier were measured on the validation set. With full florescence signals as input, the model readily learned to identify cancer cells even with only a small data subset (green curve). In other settings, performances increased linearly as the data grew exponentially.

Although our dataset already contains 25000–30000 of cells from each class, the broad heterogeneity of cell morphology may not yet be fully captured. To evaluate the impact of additional training data on cancer cell classification, the training set were artificially down-sampled to 5%, 10%, 20%, and 50% of the original size to monitor the gain in performance as the training set size grows. Figure 11 shows that the performance readily saturate with just 5% of the training data if fluorescence signals were provided as input. On the other hand, under realistic settings where brightfield images are the main source of information, cancer cell classification performance increased steadily and linearly as the size of the data grew exponentially. This strongly suggested that the model will benefit from even more training cell images.

## Usage Notes

The detailed instruction for reproducing our work was described in the directory detection and classification of our Github.

## Data Availability

All code used in this experiment was written in Python3 and could be publicly accessed at https://github.com/cmb-chula/CancerCellVision-CCA. The code is based on PyTorch^41^ and MMDetection^32^.
